# Spatial–temporal distribution of *Anopheles* larval habitats in Uganda using GIS/remote sensing technologies

**DOI:** 10.1186/s12936-018-2567-z

**Published:** 2018-11-12

**Authors:** Ryan Tokarz, Robert J. Novak

**Affiliations:** Global Health, University of South Florida, College of Public Health, 12901 Bruce B. Downs Blvd, Tampa, FL 33612-3805 USA

**Keywords:** Mosquito control, Mosquito surveillance, Autocorrelation, Moran’s *I*, Remote sensing

## Abstract

**Background:**

*Anopheles* mosquitoes impose an immense burden on the African population in terms of both human health and comfort. Uganda, in particular, boasts one of the highest malaria transmission rates in the world and its entire population is at risk for infection. Despite the immense burden these mosquitoes pose on the country, very few programmes exist that directly combat the issue at the vector control level and even fewer programmes focus on the vector in its most vulnerable juvenile stages. This study utilizes remote sensing techniques and spatial autocorrelation models to identify and prioritize the most prolific Anopheline larval habitats for control purposes in a rural community in Uganda.

**Methods:**

A community-based mosquito surveillance programme was developed and implemented in Papoli Parish in Eastern Uganda over a 4-month period. Each day, a trained field team sampled the larval habitats of *Anopheles* mosquitoes within the population-dense areas of the community. Habitats and their productivity were identified and plotted spatially on a daily basis. Daily output was combined and displayed as a weekly habitat time-series. Additional spatial analysis was conducted using the Global and Anselin’s Local Moran’s *I* statistic to assess habitat spatial autocorrelation.

**Results:**

Spatial models were developed to identify highly significant habitats and dictated the priority of these habitats for larval control purposes. Weekly time-series models identified the locations and productivity of each habitat, while Local Moran’s *I* cluster maps identified statistically significant clusters (Cluster: High) and outliers (High Outlier) that were then interpreted for control priority. Models were stitched together in a temporal format to visually demonstrate the spatial shift of statically significant, high priority habitats over the entire study period.

**Discussion:**

The findings show that the spatial outcomes of productive habitats can be made starkly apparent through initial habitat modelling and resulting time-series output. However, mosquito control resources are often limited and it is at this point that the Local Moran’s *I* statistics demonstrates its value. Focusing on habitats identified as Cluster: High and High Outlier outputs allow for the identification of the most influential larval habitats. Utilizing this method for malaria control allows for the optimization of control resources in a real time, community driven, fashion, as well as providing a framework for future control practices.

## Background

*Anopheles* mosquitoes impose an immense burden on the African population in terms of both human health and comfort. Mosquitoes within this genus are responsible for the transmission of the malaria parasite, not only on this continent, but on a global scale. African malaria is driven by species located within the *Anopheles gambiae* complex. The namesake of this complex, *An. gambiae,* is an extremely anthropophilic species that feeds almost primarily on humans and serves as the continent’s primary malaria vector [1]. This tropical species’ range spans the majority of sub-Saharan Africa, as well as Madagascar, even making its way into the continent’s southern-most countries during the warmer summer months [2, 3]. This species is of the utmost concern and considered one of the most effective and efficient malaria vectors in the world [4, 5]. In addition to *An. gambiae,* several other Anopheline species/complexes contribute to the continent’s malaria burden. These include *Anopheles arabiensis*, *Anopheles funestus*, *Anopheles melas*, *Anopheles* *merus*, *Anopheles* *moucheti* and *Anopheles* *nili* [2].

Within the African continent alone, these *Anopheles* vectors were responsible for 627,000 deaths in 2012. This accounted for approximately 80% of all malaria deaths for the year [6]. Three years later, in 2015, the region of sub-Saharan Africa was home to 90% of all malaria cases, and contributed to 92% of the estimated 438,000 deaths [7, 8]. Uganda, in particular, shoulders a significant portion of this burden. This small East African nation boasts one of the highest malaria transmission rates in the world and its entire population is at risk for infection [9, 10]. However, despite the immense burden these mosquitoes pose on the country, very few programmes exist that directly combat the issue at the vector control level and even fewer programmes focus on the vector in its most vulnerable juvenile stages.

The common approach to malaria control occurs with the domestic treatment of adult vectors and an emphasis on timely healthcare [11–14]. This approach is easily implemented and demonstrates quick results, though targets only a small portion of the mosquito population and only at a particular point in time. Malaria vector control is optimally done at the larval level where the *Anopheles* mosquito is in its most concentrated, immobile, and accessible state (CIA approach) [15]. Control in this manner allows for often scarce resources to provide the highest possible impact. Habitats of significance are identified by trained field surveillance teams and then treated accordingly with the appropriate larvicide.

This approach for malaria reduction and eradication has long proven an effective measure against the pathogen. Prior to the introduction of the potent insecticide dichlorodiphenyltrichloroethane (DDT), and a shift to adult control strategies, this method was predominant in anti-malarial campaigns [16]. In fact, Fred Soper utilized similar techniques in eradicating Africa’s most potent vector, *An. gambiae,* after it gained a foothold in Brazil [17, 18]. Larval control should optimally be implemented in the same fashion as Dr. William Gorgas’ successful anti-malaria campaign that allowed for the construction of the Panama Canal. This approach assimilated larval control into an integrated approach with synergistic interventions such as environmental modifications, screened housing, bed nets, drugs and quarantine [15]. The addition of current domestic adult treatment techniques to this array of control practices will only optimize the desired results.

One of the biggest issues preventing the establishment of such larval control operations is the inadequate resources often encountered in Uganda and other African countries [15]. The entire region of sub-Saharan Africa contains only a small number organized programmes for controlling mosquito populations, and these are often implemented only during epidemic periods [19]. It is safe to say that such programmes are overlooked or discounted based on the costs that they incur both monetarily and in time. This was very much the case in Papoli Parish, Uganda where this study took place.

An examination of economic costs for larval source management was conducted in Mbita, Kenya. This city lies just under 200 km from Papoli and embodies many of the same environmental characteristics. This study found the cost per person protected per year using such a programme to range from $1.94 to $2.50 USD depending on the larvicide formulation [20]. This cost is certainly not the same cost that would be experienced within Papoli Parish, a significantly more condensed and less populated area; however, it does illustrate the financial burden that a larval control programme can place on a community.

In an effort to combat the high cost related to larval control a statistically significant approach was taken to spatially identify the habitats of most concern at a specific point in time, and prioritize these habitats for treatment. The idea of randomly dispersed larval populations and homogenous habitat productivity and structure is not realistic when evaluating the larval composition of a community [21]. In contrast, habitat productivity is far from uniform, as certain habitats contribute largely to the overall mosquito population [21, 22]. Typically, mosquito larval dispersion is not evenly distributed or even random. It is instead considered contagious, as populations tend to aggregate in more favourable areas within the environment of the habitat [23]. This is especially true in respect to seasonal rain changes, which drive the creation and removal of habitats within a given area at a given point in time [21]. As a consequence, the management of small proportions of these clustered aquatic mosquito habitats can result in large proportional reductions in total productivity, while at the same time allowing for the optimal utilization of scarce control resources. A theoretical model depicting this technique found that with coverage of only 30% of habitats, the total productivity of an area could be reduced by 70%, and the malaria incidence in intermediate transmission areas could be similarly impacted with a reduction of 66% [21]. Identifying and targeting these particularly productive habitats can result in effective larval interventions while utilizing the minimum amount of resources [21].

The statistically significant identification and prioritization of such habitats can be completed in an easy to interpret, spatial manner, using a Moran’s *I* and Local Moran’s *I* statistic to measure spatial autocorrelation. Spatial autocorrelation refers to the correlation of the values of variable with itself through space [24]. These spatial analysis tools can be utilized to generate positive autocorrelation, which indicates clustering of similar values of a variable in close geographical range, negative autocorrelation, which indicates dissimilar values geographically nearby in space, or randomness, which represents no autocorrelation whatsoever within a dataset [24].

In terms or larval productivity for control purposes, highly productive sites located in close spatial proximity to other highly productive habitat sites are considered positively autocorrelated, while these same high values spatially located among low values are negatively autocorrelated. Though of less interest for malaria control, this same concept is true with habitats of low productivity in close spatial proximity to other low productivity sites, and low values spatially located among high values. Identification of such sites using this autocorrelation analysis becomes quite advantageous based on the contagious nature of the mosquitoes themselves.

The Moran’s *I* statistic, as identified by Moran in 1950 [25] measures the autocorrelation of a variable of interest based on the location and values present to represent a pattern globally. Moran’s *I* analysis results in a score ranging from − 1 to 1. A score near 0 indicates randomness, a score near 1 indicates clustering, and a score near − 1 indicates dispersion [26]. This statistic serves as an average for the entire dataset and therefore cannot identify specific habitats or areas of significance. Utilizing this approach; however, is quite useful in providing evidence of clustering within a dataset. Once this is established, a more focused local form of this statistic can be utilized to identify specific areas for which priority should be given for control practices.

For mosquito surveillance purposes, a local indicator of spatial association (LISA) should be used. A LISA is described by Anselin [27] to satisfy the following requirements:

1. The LISA for each observation gives an indication of the extent of significant spatial clustering of similar values around that observation.
2. The sum of LISAs for all observations is proportional to a global indicator of spatial association [27].

The preferred LISA for analysing habitat for mosquito control is the Anselin’s Local Moran’s *I* statistic. This Local Moran’s *I* statistic will generate visual representation of productive mosquito habitat clusters (positive autocorrelation). These clusters represent sets of adjoining locations for which the LISA is significant [27]. Additionally, this statistic identifies negative autocorrelation, or spatial outliers. The generation of these outliers allows for the identification of statistically significant dissimilar habitat productivity values in space. This is important, as it allows for highly productive habitats in close proximity to low or unproductive habitats to be spatially identified. These clusters and high outliers can then be prioritized for mosquito control practices.

This concept was demonstrated within the community of Papoli Parish, Uganda. A surveillance programme was initiated within the community to illustrate the ability to identify high priority habitats and clusters within a community setting in a temporal fashion. The resulting output not only identified crucial habitats for malaria control, but it also visually depicted the spatial change of these habitats over time. The use of these models can be utilized short-term for real time control practices, and over the long term to predict similar habitat distributions when conditions and seasonality are similar.

## Methods

### Study area

**Fig. 1:**
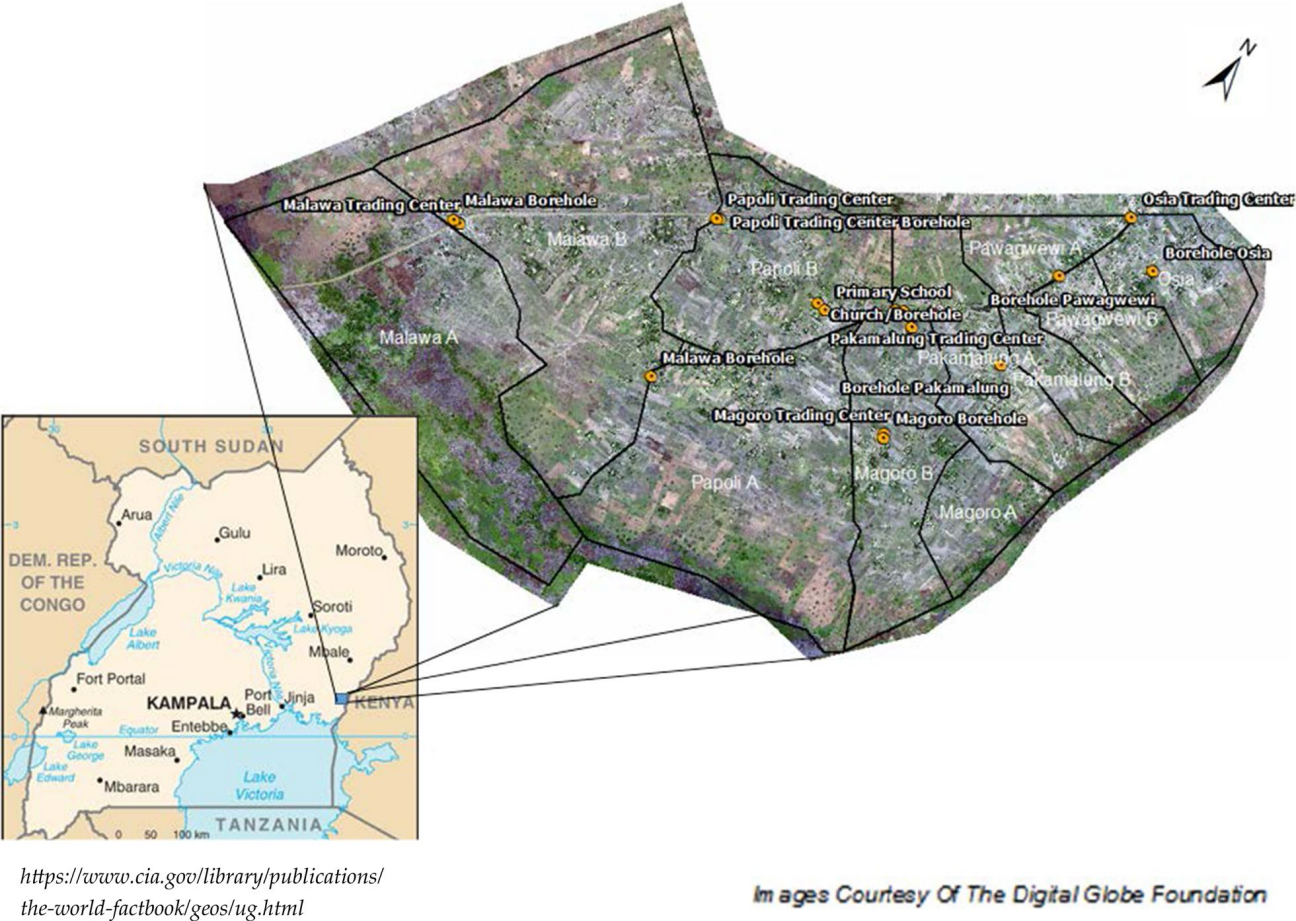
Map of Uganda with Papoli Parish, its 11 villages, and key community landmarks highlighted

The community of Papoli Parish served as the study site for this research (Fig. 1). Papoli Parish is a rural agricultural community located in eastern Uganda. The parish is located within the Tororo district and lies within Iyolwa sub-county and west Budama County; approximately 16 km from the city of Tororo.

Papoli consists of 11 zones (villages) well-suited for mosquito production. The southern boundary is comprised of a small river, the Malaba River, from which a large swamp is induced. This swamp spans the entire southern boundary. The southwestern-most boundary of Papoli is an uninhabitable swamp, also a byproduct of the Malaba River. The main highway between the cities of Jinja and Tororo runs east to west through the community and is lined on both sides by deep, water-retaining ditches which are well documented to be prolific mosquito larval habitats [4, 28, 29]. The community boundary extends slightly west beyond the main highway where the majority of land in is utilized for agriculture. Flooded rice fields of varying agricultural stages are intermittently distributed between both the northern and southern borders. Across the eastern border is an additional swamp and a small, often dry, tributary of the Malaba River.

Papoli’s interior is dominated by agriculture and family dwellings. Livestock tracks, farmed fields in various stages, open water storage, and agriculturally created standing water pools all provide optimal breeding sites for the *Anopheles* mosquito in this region [28].

### Surveillance area development

Papoli Parish is an expansive community with a multitude of possible oviposition habitats for both vector and nuisance mosquito populations. The budget for this project allowed for four full time field surveillance team members. It was not feasible to expect these team members to cover the entirety of the community within a reasonable time period. As a result, the project aimed to protect the largest number of community members with the resources available.

**Fig. 2:**
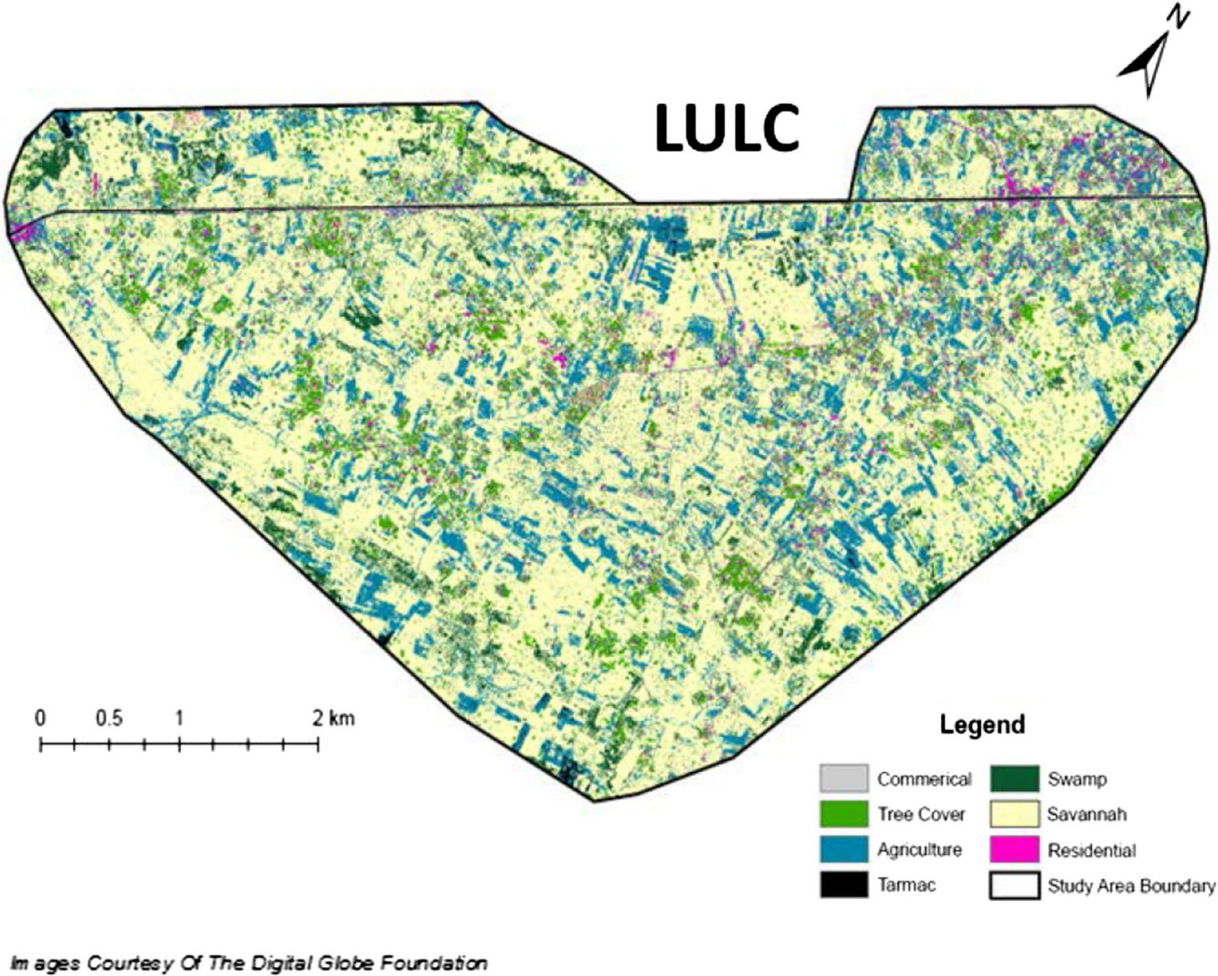
Land Use Land Cover (LULC) Model of study area within Papoli Parish

A high-resolution multispectral QuickBird-2 satellite image was obtained from Digital Globe via the Digital Globe Imagery Grant. This image was edited from its full extent to depict Papoli, Parish and its closely surrounding areas. A supervised Land Use Land Cover (LULC) was generated using this imagery within ArcGIS 10.3.1 software to identify key land cover features within the community (Fig. 2). The LULC output was utilized to pinpoint the areas of highest population density within the Parish. These highly populated areas would be further manipulated using ArcGIS software to develop our surveillance area.

**Fig. 3:**
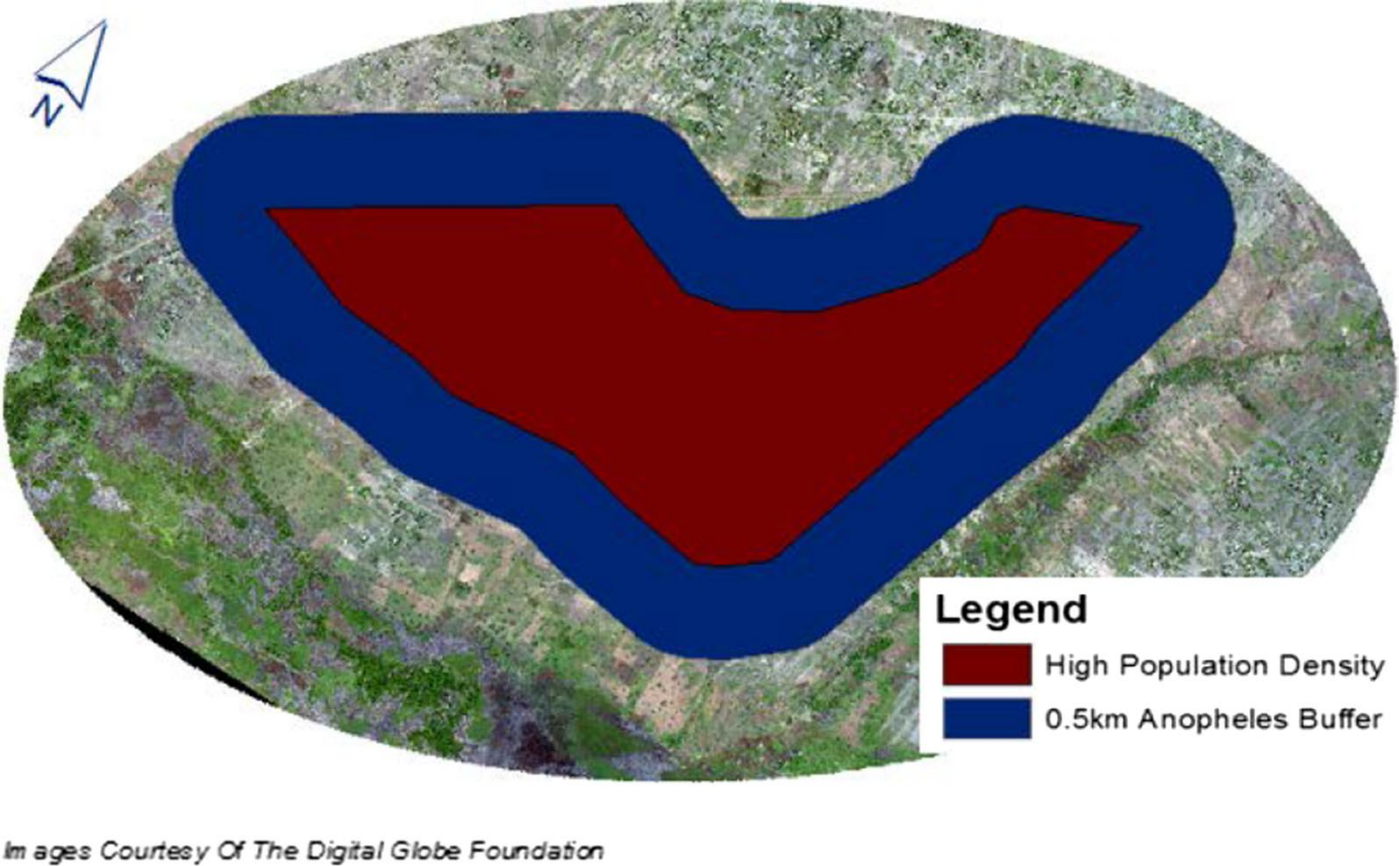
The study area within Papoli composed of the densely populated region and a 0.5 km *Anopheles gambiae* flight buffer

A polygon was created within the ArcGIS software to encompass the densely populated region. A 0.5 km buffer polygon was then developed around the population density polygon (Fig. 3). This distance of 0.5 km was strategically chosen based on the flight range of an unfed *An. gambiae*, which has been identified to be an extreme concern for malaria transmission within the region [30].

**Fig. 4:**
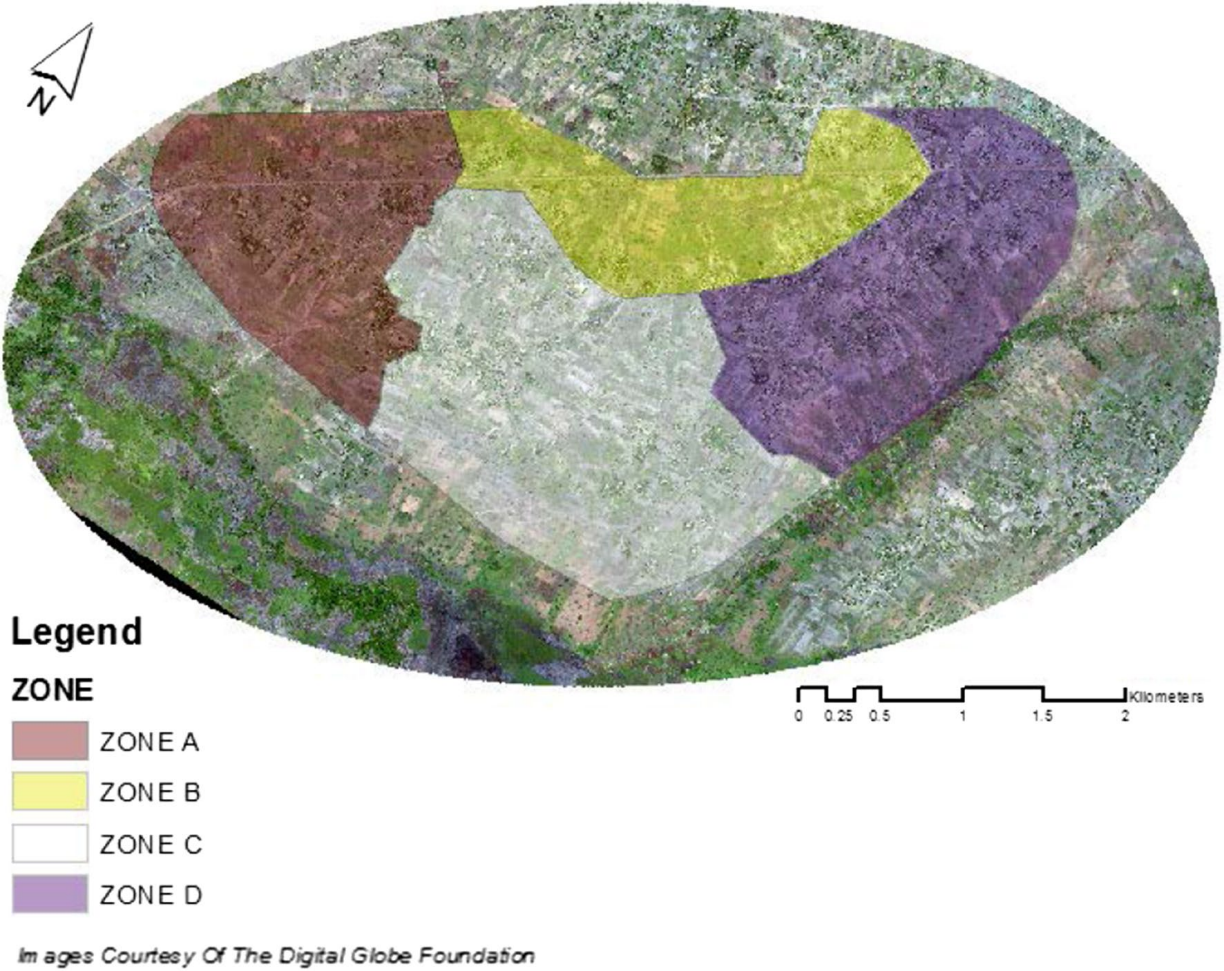
Established surveillance zones within the study area of Papoli Parish

The area contained within the two newly created polygons would serve as the surveillance area. Separation of the surveillance area into four separate surveillance zones was later determined based on environmental makeup and known local landmarks and paths (Fig. 4). These boundaries were determined with the help of the field team members who were to survey them, as well as with the knowledge of local leaders.

### Larval surveillance

Surveillance took place over a 4-month period beginning in March and ending in July of 2016. Qualified members of the Papoli community were identified and trained to properly identify all potential mosquito larval habitats within the community. These trained personnel were each assigned a specific surveillance zone and together encompassed the larval surveillance field team.

Each day 1/5 of each team member’s surveillance zone was surveyed in respect to all possible mosquito larval habitats. Each identified site was marked using a GPS unit and given its own respective waypoint identifier prior to sampling. Sites eliciting positive larval results were highlighted, and the resulting larvae captured in Whirl–Pak bags labelled in respect to the site at which they were obtained.

At the conclusion of each day, all data was returned to the project manager for processing. Habitat waypoints from each were uploaded to the field computer and converted to .kml files before storage in a spatially referenced database. All positive Whirl–Paks were emptied and the larval contents identified for specimens of the *Anopheles* genus. The resulting count data was stored by habitat in a .csv Excel database entitled Larval Surveillance Data. Each waypoint was given a unique habitat identification number (habitat ID) during its initial sampling. If sampled on an additional occasion, its original habitat ID was utilized.

### Rainfall monitoring

Surveillance was strategically initiated during the final weeks of Papoli’s dry season in an effort to map the spatial change of larval habitats during the community’s dry, rainy, and transitional periods. Rainfall served as the main water source for agriculture within the community and any irrigation that occurred was driven by rainfall events.

Rainfall has long been associated with an increase in mosquito production [4, 23]. Rainfall results in an increase in near-surface humidity, which directly influences the mosquito life cycle by increasing mosquito flight activity, oviposition, and the resulting host-seeking behaviour [31]. An increase in abundance and variety of aquatic habitats available for oviposition, and the subsequent larval progeny has also been demonstrated to result from rainfall [31–33].

This variable is not uniform in its impact for all mosquito species, however, strong correlations have been shown with the key malaria vectors [34]. For example, due to the temporary nature of preferred habitats, increased rainfall is associated with an increase in *An. gambiae* [35–37]. This species serves as one of Papoli’s most potent vectors. Rainfall was monitored daily with a rain gauge placed centrally in the community.

### Habitat modelling

In order to generate an idea of the *Anopheles* habitat distribution within Papoli, visually descriptive spatial maps were generated as daily data was obtained. These maps were combined into weekly maps that spatially depicted the entirety of each week’s surveillance data. Weekly maps were stitched together over time in a Time-Series fashion to visually depict the location and count of *Anopheles* larva throughout the entirety of the surveillance programme.

Each week, all daily .kml files were merged to a single weekly .kml using .kml merging software. The KML to Layer function within the ArcGIS software was then utilized to convert the .kml file to a workable point shapefile, which was saved and labelled to its respective week. The Larval Surveillance Data Excel database was then joined to the spatially represented data within the ArcGIS software. Once joined, graduated symbols and individual colors depicting larval count were created to represent habitats of varying productivity. Graduated symbols and individual colors were demarcated by a count of 5 *Anopheles* mosquitoes, beginning at 0 and ending with a > 15 category. Habitat productivity was ultimately categorized into 1 of 5 categories; 0, 1–5, 6–10, 11–15, > 15. As weekly models accumulated, the temporal change of habitats and their productivity becomes apparent. These time-series models are not statically significant, but do provide stark visuals from which the understanding of local mosquito distribution can begin.

### Spatial autocorrelation analysis

In addition to weekly productivity maps, maps displaying statistically significant clustering and outliers were generated to identify areas of high concern for *Anopheles* production. These maps were produced within ArcGIS software using the same weekly joined shapefile and .csv file utilized to produce the weekly productivity map. Spatial autocorrelation patterns were analysed and displayed using the Spatial Statistics Extension available in ArcGIS.

### Moran’s *I* spatial autocorrelation

Spatial autocorrelation patterns of *Anopheles* larval counts were first analysed on a weekly basis using an inverse distance spatial relationship with a Euclidean distance calculation via the Moran’s *I* spatial autocorrelation tool. This global statistic analyses the locations and count values of each habitat simultaneously to measure autocorrelation [25]. The strength of the correlation between the count values is estimated as a function of the distance between their respective habitats and calculated on average for the dataset [38]. The product of this analysis was used to determine clustering, randomness, or dispersion of the data.

A Moran’s *I* index output is generated and ranges from − 1 to 1. As the output approaches 1, the intensity of clustering increases. A score of 0 indicates randomness, while the closer to − 1 the index is, the more dispersed the data is it represents. In respect to habitat prioritization for malaria control, the interest is focused on clustering. This clustering indicates statically significant groupings of habitats. Clustering is indicated by a Moran’s *I* index greater than 0, a significant p-value of ≤ 0.10 (90% Confidence) and a z-score ≥ 1.65.

### Anselin’s Local Moran’s *I*

The global Moran’s *I* index output provides evidence of spatial autocorrelation within the dataset, but cannot provide specific locations of autocorrelation, as it tends to average local variations [38]. To visually display and analyse the spatial autocorrelation of the data, a local indicator of spatial association (LISA) in the form of an Anselin’s Local Moran’s *I* was employed. An Anselin’s Local Moran’s *I* was analysed on a weekly basis for *Anopheles* larval counts. This was done using an inverse distance spatial relationship with a Euclidean distance calculation via the Cluster and Outlier Analysis (Anselin Local Morans *I*) tool within the ArcGIS software.

The LISA generated from each point displays an indication of statistically significant clustering of similar or dissimilar values around that point in space [27]. The use of this Local Moran’s *I* statistic spatially depicts hot spots, cold spots, and statistically significant spatial outliers in 1 of 4 forms: Cluster: High, Cluster: Low, High Outlier, and Low Outlier. Cluster: High outputs can be considered hot spots. These areas represent highly productive habitats within close proximity to each other. A High Outlier represents a habitat that is highly productive, but surrounded by unproductive or low productivity habitats, while Low Outlier displays a low productivity habitat surrounded by highly productive habitats. Cluster: Low indicates the inverse of Cluster: High. For mosquito control purposes, the priority outputs are Cluster: High and High Outlier output, as these indicate areas of highest productivity relative to overall larval count and distance.

This identification of spatial outliers and significant hot spot clusters is the key indicator for determining habitat priority for mosquito control purposes. All identified Anopheline spatial outliers and significant hot spot clusters were modelled with a 0.5 km buffer. This buffer provides visual evidence of at risk areas from these potent malaria vectors.

## Results

### Rainfall

**Fig. 5:**
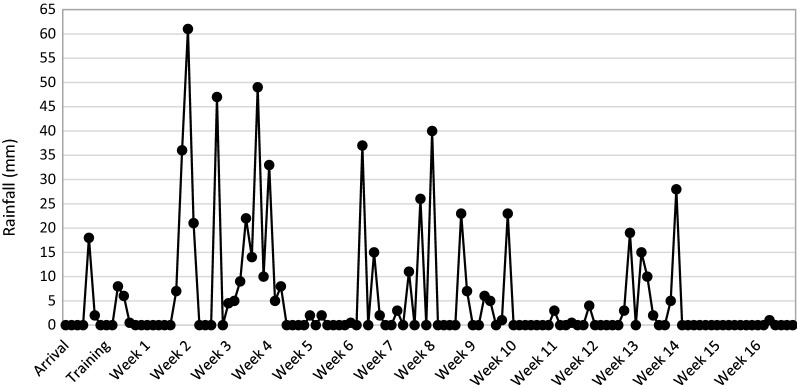
Daily rainfall by week during the study period

Rainfall was tracked and recorded daily from the week prior to arrival, through the training period, until the competition of the study (Fig. 5). Larval production generally mimicked rainfall patterns within the community. An increase in rainfall resulted in a corresponding increase of overall larval habitat count and productivity.

After a prolonged dry period, consistent rainfall began during the second week of surveillance and mosquito productivity increased shortly thereafter. Weeks 2 through 10 represented the rainy season for the community. Rainfall reduced in intensity around week 10 and was fairly scarce during weeks 10 and 11. Rainfall resumed during weeks 12–14, before essentially stopping completely until the conclusion of surveillance.

Key rainfall intervals were identified at four points in the study period: a prolonged dry period, transition into a rainy period, prolonged rainy period, and a transition into a dry period. The first interval, the prolonged dry period, was represented by the weeks prior to the beginning of the study period as well as the training and preparatory periods of surveillance. Within this time, rainfall was scarce, occurring sporadically and for short periods, with no evident pooling of water.

The heaviest rains occurred within weeks 2 through 5 of the study period. This time frame represents the transition into a prolonged rainy period and is representative of the second key interval. A slight dip in rainfall occurred for approximately 1 week around week 5, before consistent rainfall returned for weeks 6 through 10. This 5-week period represents the prolonged rainy period and the third key rainfall interval. The final interval represents the transition into a dry period and occurs from weeks 11 through the conclusion of the study period. During this time, rainfall is shown to taper significantly until essentially non-existent.

Rainfall was not statistically analysed within the parameters of the spatial outcomes; it instead provided context to the changes in potential habitat numbers and location. The undulation of the resulting habitats and their productivity was captured and displayed within spatial–temporal time-series models.

### Weekly habitat modelling

**Fig. 6:**
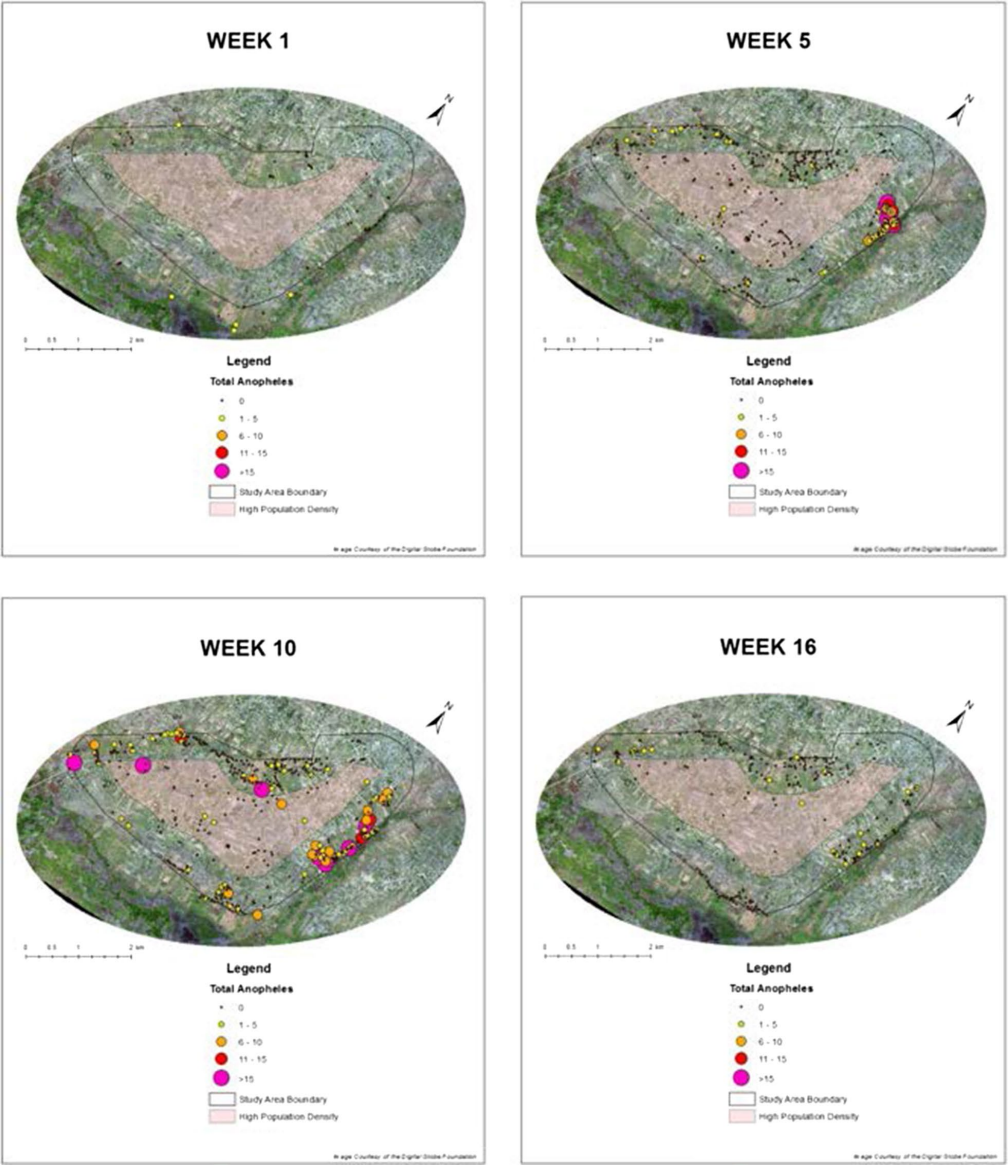
Spatial habitat models at the conclusion of each key rainfall interval: weeks 1, 5, 10, 16

Habitat models were developed on a weekly basis depicting the spatial distribution of each habitat surveyed. Weeks 1, 5, 10, and 16 are displayed to exhibit the clear variance of habitat location and productivity over time at the conclusion of each key rainfall interval identified (Fig. 6). These weekly models provided an easy to decipher visual on the distribution and larval count of productive *Anopheles* habitats. Stitching weekly models together allowed for the spatial transition of larval count per habitat to be observed over time in a time-series fashion. This information provides an initial frame of reference for optimal locations for which to conduct *Anopheles* control over the course of the surveillance programme.

Habitats were extremely scarce upon the first week of surveillance due to the long term dry conditions. This was by far the least productive week, so much so, that almost all identified productive habitats fall outside of the surveillance boundaries in the Southern swampy areas of the community (Fig. 6). These areas were surveyed after all areas within the demarcated surveillance area were surveyed and exhausted.

Consistent rains began during the early portion of week 2 and *Anopheles* productivity increased in turn during the following weeks. By week 5 clear clustering of productive habitats became apparent in the rice paddies located within the northeast region of the Parish. At the same time, positive habitats begin to appear in small numbers in the western agricultural regions of the community (Fig. 6).

As the rainy period continued into its final week (week 10), *Anopheles* larvae and habitats within the northeastern rice paddies continued to increase in abundance. At this this juncture, productive Anopheline habitats were also shown to have spread into the interior of the community, as well as western agricultural lands. Visually, week 10 elicited the most spatially diverse and highly productive habitat distribution within the study period (Fig. 6).

The rains begin to subside during weeks 1 and 11 and *Anopheles* habitats reacted similarly, slowly reducing in productivity and abundance. By the conclusion of the study, in week 16, one could observe a significant reduction in Anopheline count and productivity, and a spatial recession from the interior of the community to locations only in the agricultural outskirts (Fig. 6).

### Spatial statistical analysis

The weekly habitat and resulting time-series models were able to visually depict the locations of *Anopheles* habitat productivity and clustering over time. However, this analysis was simply visual and could not provide significant reasoning for prioritization of similarly productive habitats. This reasoning was afforded through the implementation of weekly statistical analysis of all habitat locations and productivity in relation to one another in space.

### Moran’s *I* spatial autocorrelation

The Global Moran’s *I* statistic is not meant to, and did not, provide information on specific habitat productivity or location within Papoli. Instead, this analysis provided evidence that significant clustering occurred within the community. The Global Moran’s *I* statistic was able to identify clustering in 11 of the 16 surveillance weeks. Of the 5 non-clustered weeks, 3 occurred during low count dry periods. The remaining 2 instances fell slightly outside the range of statistical significance for clustering (Table 1).

**Table 1:** Moran’s *I* Autocorrelation

Week	Moran’s Index	z-score	p-value
1	− 0.013066	0.012054	0.990382
2	0.010379	0.225019	0.821964
3	0.168173	3.883808	0.000103
4	0.629283	14.461069	0.000000
5	0.622881	16.716332	0.000000
6	0.251934	4.818493	0.000001
7	0.054189	1.466515	0.142508
8	0.058488	1.618322	0.105593
9	0.144689	4.203926	0.000026
10	0.080315	3.211992	0.001318
11	0.225571	5.321790	0.000000
12	0.105720	2.560649	0.010448
13	0.078098	3.115252	0.001838
14	0.202997	7.390563	0.000000
15	0.032872	1.138473	0.254923
16	0.017599	0.591973	0.553869

The first 2 weeks of sampling resulted in a pattern that was not significantly different than that of the random. Z-scores of 0.012054 and 0.225019 and Moran’s index outputs of − 0.013066 of 0.010379 were reported for weeks 1 and 2, respectively. This randomness can be attributed to sample size, as only 6 positive *Anopheles* sites were identified during the first week, and 7 were identified the following week. The rains began in the midst of week 2, and by week 3 enough positive habitats were identified to indicate clustering. Though only 14 sites were identified, these occurred in such a manner that clustering was evident.

The most intense clustering occurred during weeks 4 and 5. Moran’s Index and z-scores were significantly higher during this 2-week period than any other within the study sample. Week 4 displayed a z-score of 14.461069 along with a Moran’s index of 0.629283, while week 5 had slightly stronger clustering outputs with a z-score of 16.716332 and a Moran’s index of 0.622881. Significant clustering continued through week 6, which elicited outputs of 4.818493 and 0.251934 for the z-score and Moran’s index.

Slight changes occurred with the output for week’s 7 and 8, as these weeks were just outside the parameters of clustering, and instead were categorized as random. Week 7 had a p-value of 0.142508 and a z-score of 1.466515, while week 8 was even closer to clustering with a p-value and z-score barely outside the clustering parameters, 0.105593 and 1.618322, respectively. Both weeks had a Moran’s index close to 0.05.

Clustering is indicated once again during week 9 and continues in this fashion until week 15 when habitat counts significantly decreased. Week 15 generated a Moran’s index of 0.032872, a z-score of 1.138473, and a p-value of 0.254923, thus displaying randomness. Similar values indicative of randomness were generated for the final week of surveillance with outputs of 0.017599 for Moran’s index, 0.591973 z-score, and a p-value of 0.553869. Habitats scarcity during these final weeks mimicked the early weeks of surveillance as rainfall had subsided significantly by the conclusion of the surveillance period.

### Anselin’s Local Moran’s *I*

**Fig. 7:**
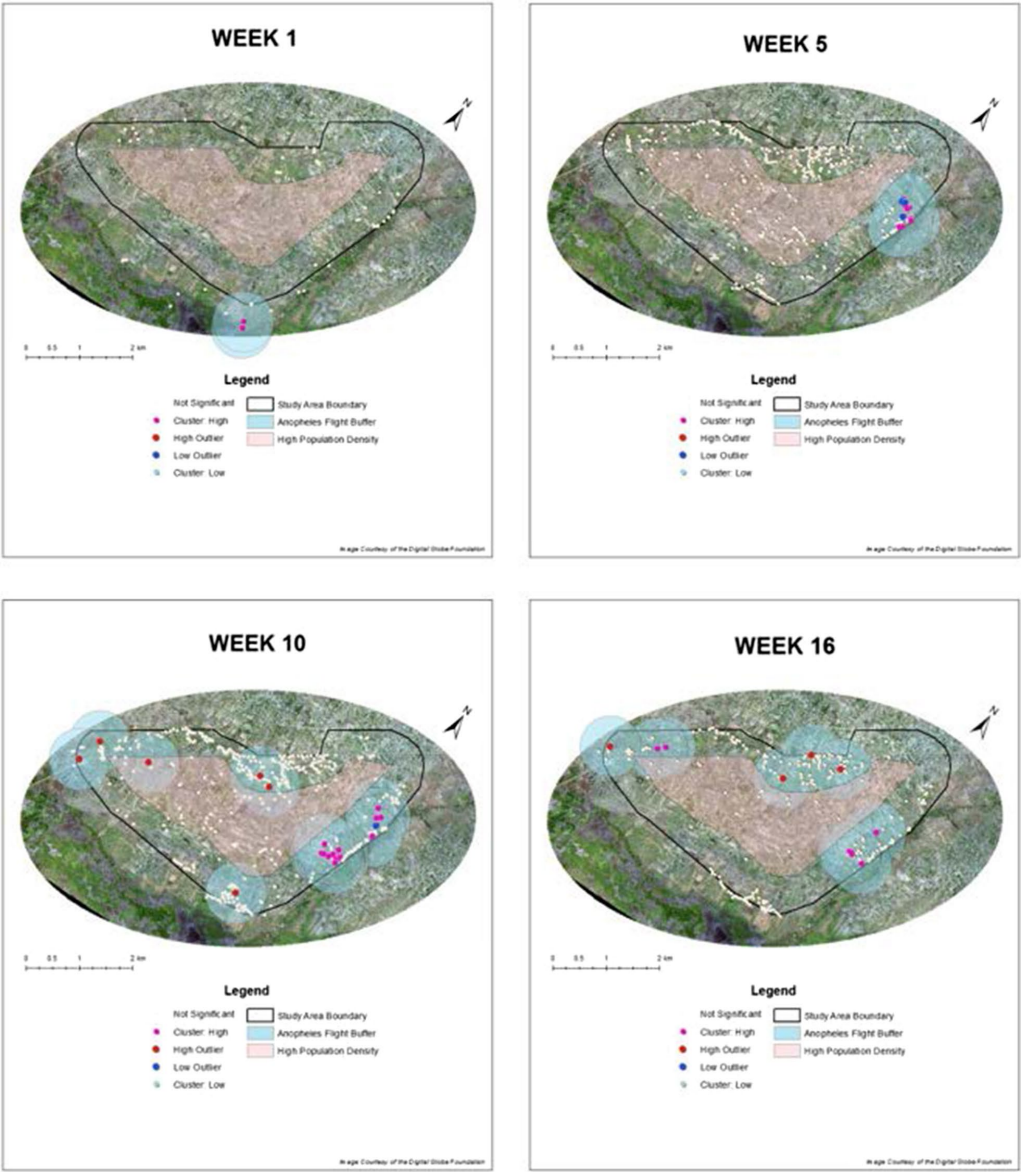
Anselin’s Local Moran’s *I* output and corresponding 0.5 km *Anopheles gambiae* flight buffer at the conclusion of each key rainfall interval: weeks 1, 5, 10, 16

In contrast to the global statistic, the Local Moran’s *I* statistic was able to determine and spatially depict statistical significance for all weeks of surveillance (Fig. 7). The global and local patterns may not always align since the global statistic decomposes into its various components within a LISA [27]. As a result, it is possible for clustering to occur on the local level, even if it was not established on the global level.

Results for the first week of sampling identify only two instances of highly productive habitat clustering. These areas occur in the southernmost part of the community outside of our surveillance area (Fig. 7). Habitats and habitat productivity are extremely limited during this period as rainfall has been extremely scarce in the previous weeks and months. Between weeks 2 and 5 clustering begins to expand and make its way to the highly productive northeastern portion of the community. From this juncture onward, this rice agricultural-dominant region is established as a key area for Anopheline production. By week 5 multiple highly productive habitat locations within close proximity to each other are identified in this area. At this same time, enough habitat productivity and diversity occur for the identification of Low Outliers to be displayed in this same area. Such Low Outlier locations are devoid of Anopheline larvae, but are closely surrounded by productive Anopheline habitats (Fig. 7).

Weeks 6 through 10 represent an established rainy period within Papoli. The Moran’s *I* output for week 10 depicts an expansion of key Anopheline habitats that result from such extended precipitation. The northeast portion of the community remains an intense area of high clustering, however, Low Outlier instances have reduced in this area, while High Outliers begin to appear more prominently in other areas of the community. The west, northwest, and southern portions of the community all display instances of High Outlier autocorrelation (Fig. 7). These areas represent isolated high *Anopheles* larval activity habitats in close proximity to a multitude of non-*Anopheles* productive habitats.

The habitat spatial output for the remaining weeks begins to reduce in number and location diversity as the rainy period subsides and a transition into a dry season begins. By week 16 the northeastern portion still elicits high clustering activity, but in a much reduced capacity and with no instances of Low Outliers. Cluster: High areas are also evident in the western portion of the community along with a single high outlier area. Additional high outliers are located in the northwestern portions during this week (Fig. 7).

## Discussion

It is fairly obvious after 4 months of surveillance and spatial mapping that the majority of Papoli’s *Anopheles* mosquitoes are originating within the northeastern portion of the community, within the rice paddy-based agriculture. Other locations of significance similarly become apparent as surveillance progresses temporally.

Visually, the spatial outcomes of productive habitats are made starkly apparent through the initial habitat modelling and resulting time-series output. In a well-funded control operation, these spatial models alone are sufficient enough information to dictate locations for treatment operations. In such an operation, each productive habitat can be identified and treated. This approach is targeted, effective, and does not waste resources by blanket treating an entire area, regardless of productivity. However, mosquito control resources are often limited. This is especially true in the rural areas of the world most impacted by the malaria burden. It is at this point that the Local Moran’s *I* statistics demonstrates its value.

The most important contribution of the Local Moran’s *I* in this context is the ability to prioritize habitats based on the spatial significance of their productivity. In instances where resources are extremely limited this statistic will identify essential areas for control by pinpointing the highest areas of production. Prioritizing control in this fashion will maximize the impact of all available resources.

Cluster: High and High Outlier outputs can be assessed as priority locations for control purposes. Cluster: High outputs are of the utmost importance and serve as the top priority for control. Habitats with this distinction are indicative of multiple sites of high larval productivity within close proximity and are most certainly contributing heavily to the *Anopheles* burden of the community. Following only slightly behind Cluster: High outputs in the hierarchy of importance for control measures are High Outlier outputs. These areas of negative spatial autocorrelation pinpoint locations, in otherwise unproductive spaces, that are significantly producing larva of these disease vectors. The ability to identify and treat these locations will make a significant impact on the *Anopheles* mosquito population of a community. And from this reduction in population, it can be inferred that malaria transmission itself will follow suit.

Low Outlier and Cluster: Low outputs also provide useful information regarding the community’s malaria vector makeup. Further analysis of these unproductive areas may provide valuable information on the characteristics of habitats that are resulting in the lack of production. This same approach can be taken with the High Outlier habitats in an effort to better understand their productivity in relation to the unproductive sites surrounding them. Integrating this knowledge into the field surveillance process can only enhance the programmes efficacy.

Other statistics for local spatial association, such as the Getis-Ord Gi* statistic and Local Geary’s *C* statistic were possible candidates for analysis, but each has a limiting factor that reduces its efficacy for use in mosquito surveillance and control. The Getis-Ord Gi* statistic is essentially an analysis of spatial association through the identification of hot and cold spots [39]. Though quite useful for identifying these areas of positive autocorrelations, this tool does not have the ability to identify negative autocorrelation, which represent our significant outliers.

Geary’s *C* is quite similar to Moran’s *I*, but functions in the inverse fashion. As opposed to measuring spatial autocorrelation, this statistic is instead a spatial measurement of dissimilarity, or negative autocorrelation. This approach can still identify both negative and positive autocorrelations, but has been consistently shown to be less powerful when compared to the Moran’s *I* on a global level [40, 41]. Additionally, a univariate local Geary’s *C*, such as that dictated by mosquito larval count, is more complex in the interpretation of a location with a significant statistic when compared to both Local Moran’s *I* and Getis-Ord [42]. Using a Moran’s *I* statistic allows for a more powerful global statistic, as well as a simple, easy to interpret output when deconstructed to the local level.

The techniques outlined serve as a template for similar surveillance and control operations. The variables and time frames can be manipulated to best fit the resources of each control programme. Daily modelling and subsequent control activities could be implemented to combat productivity on a more efficient basis. This approach would prevent emergence of late instar and pupal specimens that were identified during the early week portions of surveillance. Similarly, pupae, as opposed to total larvae could serve as the dependent variable for model development. Pupal count is positively associated with habitat stability and productivity and may serve as a better indicator of habitat of significance [43].

The true driving force behind the success of the prioritization of habitats is field surveillance. Spatial analysis is only as effective as the data it utilizes. A field team must be well trained and disciplined in all aspects of habitat identification and sampling. Human error is to be expected, as all habitats may not be able to be identified and productivity may not always be exactly accurate. However, the better trained and more skilled each field team member is the more effective habitat prioritization in this fashion will be.
